# Cadmium-Induced Kidney Injury in Mice Is Counteracted by a Flavonoid-Rich Extract of Bergamot Juice, Alone or in Association with Curcumin and Resveratrol, via the Enhancement of Different Defense Mechanisms

**DOI:** 10.3390/biomedicines9121797

**Published:** 2021-11-30

**Authors:** Santa Cirmi, Alessandro Maugeri, Antonio Micali, Herbert Ryan Marini, Domenico Puzzolo, Giuseppe Santoro, Jose Freni, Francesco Squadrito, Natasha Irrera, Giovanni Pallio, Michele Navarra, Letteria Minutoli

**Affiliations:** 1 Department of Chemical, Biological, Pharmaceutical and Environmental Sciences, University of Messina, 98166 Messina, Italy; scirmi@unime.it (S.C.); amaugeri@unime.it (A.M.); 2 Department of Pharmacy-Drug Sciences, University of Bari “Aldo Moro”, 70125 Bari, Italy; 3 Department of Biomedical and Dental Sciences and Morphofunctional Imaging, University of Messina, 98125 Messina, Italy; amicali@unime.it (A.M.); puzzolo@unime.it (D.P.); giuseppe.santoro@unime.it (G.S.); freni.jose.89@gmail.com (J.F.); 4 Department of Clinical and Experimental Medicine, University of Messina, 98125 Messina, Italy; hrmarini@unime.it (H.R.M.); fsquadrito@unime.it (F.S.); nirrera@unime.it (N.I.); lminutoli@unime.it (L.M.)

**Keywords:** kidney, cadmium, flavonoids, nutraceuticals, bergamot juice, resveratrol, curcumin, oxidative stress, inflammation, apoptosis

## Abstract

Cadmium (Cd) represents a public health risk due to its non-biodegradability and long biological half-life. The main target of Cd is considered the kidney, where it accumulates. No effective treatment for Cd poisoning is available so that several therapeutic approaches were proposed to prevent damages after Cd exposure. We evaluated the effects of a flavonoid-rich extract of bergamot juice (BJe), alone or in association with curcumin (Cur) and resveratrol (Re), in the kidney of mice exposed to cadmium chloride (CdCl_2_). Male mice were administered with CdCl_2_ and treated with Cur, Re, or BJe alone or in combination for 14 days. The kidneys were processed for biochemical, structural and morphometric evaluation. Cd treatment significantly increased urea nitrogen and creatinine levels, along with *tp53*, *Bax*, *Nos2* and *Il1b* mRNA, while reduced that of *Bcl2*, as well as glutathione (GSH) content and glutathione peroxidase (GPx) activity. Moreover, Cd caused damages to glomeruli and tubules, and increased *Nrf2*, *Nqo1* and *Hmox1* gene expression. Cur, Re and BJe at 40 mg/kg significantly improved all parameters, while BJe at 20 mg/kg showed a lower protective effect. After treatment with the associations of the three nutraceuticals, all parameters were close to normal, thus suggesting a new potential strategy in the protection of renal functions in subjects exposed to environmental toxicants.

## 1. Introduction

Cadmium (Cd) is a non-essential metal present at position 7 on the substance priority list of the Agency for Toxic Substances and Disease Registry (ATSDR 2019). Mainly, it is an environmental and industrial toxicant, derived from incineration, refining, mining, and fossil fuel combustion. Environmental exposure to Cd is progressively increasing, owing to the wide use of Cd-containing goods in industrialized countries, and it represents a major public health risk due to its non-biodegradability as well as to its long biological half-life (10–30 years) [1].

Environmental Cd may accumulate in many organs, such as liver, lung, testes, and bones. However, the main target of Cd is considered the kidney, particularly the proximal tubules and renal glomeruli [2,3], where it accumulates, owing to the absence of a specific mechanism for elimination. Indeed, after chronic exposure to Cd, approximately 50% of the total body stores accumulate in the kidneys [4], making them particularly susceptible to Cd-mediated nephrotoxicity.

After having been absorbed by the organism, Cd binds to metallothionein, a cysteine-rich, low-molecular weight metal-binding protein [5]. The complex Cd-metallothionein is filtered into the Bowman’s space by the glomerular capillaries and internalized by the proximal tubule cells. The complex is then degraded by lysosomes to release Cd [6]. In the cytoplasm, the release of Cd is known to produce reactive oxygen species (ROS), cause glutathione (GH) exhaustion, lipids peroxidation, protein crosslinking, and inflammation. As a result, an accumulation of pro-inflammatory cytokines and kidney cell death occur, leading to kidney toxicity [6,7].

Further mechanisms of Cd renal toxicity have been also described: among them, mitochondrial damage [8], cellular death, in particular apoptosis induction [9], disruption of cadherin-mediated cell-cell adhesion in the proximal tubule cells [10], and stimulation of the inflammation pathways [11] were observed.

Currently, there is no effective treatment for Cd poisoning. The principal therapeutic protocol involves employment of metal chelator, although they cause several undesirable effects, such as redistribution/translocation of metals and other serious toxic manifestations [12]. This caught the interest of scientists who have sought for an effective remedy from natural sources, that are less prone to toxic effects. In recent years, several natural products have been proposed to prevent structural and functional damages following environmental or experimental Cd exposure, with particular attention to the protective functions of plant-derived antioxidants such as carnosic acid [13], chocolate [14], grape seed procyanidin [15], alphalipoic acid [16], flavocoxid [17] and myo-inositol [18].

In the last decades, bergamot juice (BJ), obtained from *Citrus bergamia* Risso et Poiteau (bergamot) fruits, has attracted the attention of scientist on antioxidant natural products [19,20]. Indeed, it has been shown that BJ and its flavonoid-rich fraction (BJe) exert several biological activities, among which anti-cancer [21], anti-infective [22,23], hypolipemic and hypoglycemic [24], neuroprotective [25,26], antioxidant [27,28] and anti-inflammatory effects [29,30,31,32]. Moreover, very recently, we showed that BJe reduces the testicular damage induced by Cd through a mechanism involving its anti-inflammatory and anti-apoptotic activities [33].

Curcumin (Cur) or diferuloylmethane (1,7-bis(4-hydroxy-3-methoxyphenyl)-16-heptadiene-3,5-dione) is a hydrophobic polyphenol extracted from the rhizome of *Curcuma longa* L., commonly known as turmeric. Its protective effect on kidney damage, associated with its antioxidant, anti-inflammatory and anti-tumorigenesis properties, has been broadly described in several experimental models [34,35].

Resveratrol (Re; 3,5,4′-trihydroxy-trans-stillbene), is a naturally occurring polyphenol found mainly in in the skin of grapes, in berries, and peanuts. Re has been studied for its pharmacological effects, including antioxidant, anti-inflammatory, immunomodulatory, hepatoprotective, anti-cancer, anti-atherosclerotic, and anti-diabetic properties [36,37,38,39].

On these bases, the present study was designed to investigate the effects of the nutraceuticals BJe, Cur, and Re, alone or in association, in a murine model of Cd-induced kidney damage.

## 2. Materials and Methods

### 2.1. Ethical Approval

All the experiments were conducted in conformity with the Italian Guidelines for Care and Use of Laboratory Animals (D.L.116/92) and with the European Directive (2010/63/EU), as well as in compliance with the ARRIVE (Animal Research Reporting In Vivo Experiments) guidelines. The study was approved by the National Ethics Committee for Research Animal Welfare of the Italian Ministry of Health (authorization no. 112/2017-PR, 2 February 2017) and by the Institutional Animal Care and Use Ethic Committee of the University of Messina (OPBA, #820/2016, 2 September 2016).

### 2.2. Drugs and Chemicals

The BJe used in this research was already used in our previous research [21,33,40]. Its quali-quantitative analysis showed that neohesperidin (94.00 mg/g), naringin (92.4 mg/g), melitidin (56.2 mg/g), hesperetin (51.9 mg/g), neoeriocitrin (48.6 mg/g) and naringenin (27.3 mg/g) were the most abundant flavonoids. Cadmium chloride (CdCl_2_), Cur and Re were purchased from Sigma-Aldrich Srl (Milan, Italy). All other chemicals not listed were commercially available reagent grade.

### 2.3. Experimental Protocol

**Figure 1:**
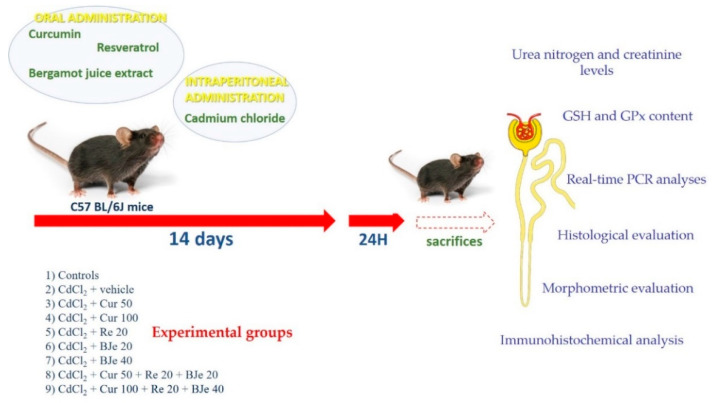
Graphical scheme of the study design. The dose of CdCl_2_ was 2 mg/kg, while those of Cur, Re and BJe are expressed as mg/mL.

A total of 119 adult male C57 BL/6J mice, weighing 25–30 g, were acquired from Charles River Laboratories Italia Srl (Calco, LC, Italy) and housed at the animal facility of the School of Medicine of the University of Messina, Messina, Italy. The animals were fed with a standard diet *ad libitum* with free access to tap water under a 12-h light/dark cycle. The animals were randomly included in 17 groups of 7 mice each. Nine groups were used as control (i) 0.9% NaCl (vehicle); (ii) corn oil (vehicle); (iii) Cur (50 mg/kg); (iv) Cur (100 mg/kg); (v) Re (20 mg/kg); (vi) BJe (20 mg/kg); (vii) BJe (40 mg/kg); (viii) Cur (50 mg/kg) + Re (20 mg/kg) + BJe (20 mg/kg); (ix) Cur (100 mg/kg) + Re (20 mg/kg) + BJe (40 mg/kg), while 8 groups were treated as follows: (i) CdCl_2_ (2 mg/kg) + vehicle; (ii) CdCl_2_ + Cur (50 mg/kg); (iii) CdCl_2_ + Cur (100 mg/kg); (iv) CdCl_2_ + Re (20 mg/kg); (v) CdCl_2_ + BJe (20 mg/kg); (vi) CdCl_2_ + BJe (40 mg/kg); (vii) CdCl_2_ + Cur (50 mg/kg) + Re (20 mg/kg) + BJe (20 mg/kg); (viii) CdCl_2_ + Cur (100 mg/kg) + Re (20 mg/kg) + BJe (40 mg/kg). CdCl_2_, Re, and BJe were dissolved in 0.9% NaCl; Cur was dispersed in corn oil. The oral administration of Cur, Re, and BJe and intraperitoneal (i.p.) challenge with CdCl_2_ was performed for 14 days. The doses of all substances employed were selected in accordance with previous studies [17,18,21,33,40,41,42,43,44,45,46]. Twenty-four hours after the last treatment, all mice were sacrificed with an overdose of ketamine and xylazine (75/10 mg/kg i.p. each) and bilateral nephrectomies were performed. The kidneys were processed for molecular, histological, and immunohistochemical evaluation. A graphical scheme of the study design is reported in Figure 1.

### 2.4. Urea Nitrogen and Creatinine Levels Quantification

After clotting, blood samples were centrifuged and urea nitrogen was quantified by a colorimetric kit (Roche Diagnostics GmbH, Mannheim, Germany), following manufacturer’s guidelines [17]. Creatinine levels were evaluated by an enzymatic method with an automatic analyzer (Roche Diagnostics GmbH).

### 2.5. Determination of Glutathione (GSH) and Glutathione Peroxidase (GPx) Content

GSH content was measured in the kidneys of all groups according to Ellman’s (1959) method, as recommended by Gong and co-workers [47]; while, glutathione peroxidase (GPx) was evaluated according to Flohé and Günzler [48], as detailed by Manna and collaborators [49].

### 2.6. Real-Time PCR Analyses

Total RNAs from kidney samples from animals of all challenged groups were extracted with the TRIzol LS reagent (Invitrogen, Carlsbad, CA, USA) according to the manufacturer’s guidelines. Then, 2 µg of RNA from each sample were reverse transcribed into cDNA using the High-Capacity cDNA Archive Kit (Applied Biosystems, Life Technologies, Foster City, CA, USA). The mRNA expression was evaluated by Real-time PCR, as previously described [21]. The sequences of primer employed for the real-time PCR analyses are listed in Table 1. The Real-time PCRs were carried out in 20 µL reactions containing 1xSYBR^®^ Select Master Mix (Applied Biosystems), 0.2 µM of primers, and 25 ng RNA converted into cDNA. The analyses were performed in triplicate in a 96-well plate using a 7900HT Fast Real-Time PCR System (Applied Biosystems). Data were collected and analyzed using the 2^−ΔΔCT^ relative quantification method with β-actin (*Actb*) used as endogenous control. The values are presented as fold changes relative to the control tissues.

### 2.7. Histological Evaluation

The kidneys were fixed in freshly prepared Bouin solution, dehydrated in graded ethanol, cleared in xylene, and embedded in paraffin (Paraplast, SPI Supplies, West Chester, PA, USA). Five-micrometer sections were stained with hematoxylin and eosin (HE) and periodic acid-Schiff (PAS). The slides were photographed with a Nikon Ci-L (Nikon Instruments, Tokyo, Japan) light microscope by a digital camera Nikon Ds-Ri2, and saved as Joint Photographic Experts Group (JPEG) with the software Adobe Photoshop 2021 (Adobe, San Jose, CA, USA).

### 2.8. Immunohistochemical Analysis for IL-1β and Nrf2

Paraffin-embedded 5-μm sections, derived from the same specimens used for histological evaluation, were assembled on Polysine slides (Thermo Fisher Scientific, Waltham, MA, USA), cleared in xylene and rehydrated in ethanol. Antigen retrieval was achieved with citrate buffer (pH 6.0) and endogenous peroxidase was stopped with 0.3% H_2_O_2_ in phosphate buffer saline (PBS). Primary antibodies IL-1β (1:250, Santa Cruz Biotechnology, Dallas, TX, USA) and Nrf2 (1:150, St. John’s Laboratory, London, UK) were incubated overnight at 4 °C in a moisturized chamber. The day after, the secondary antibodies (Vectastain, Vector, Burlingame, CA, USA) were added and 3,3′-Diaminobenzidine (DAB) (Sigma-Aldrich) was used to visualize the reaction. The sections were counterstained with Mayer’s hematoxylin. Appropriate positive and negative controls were used in each test. Slides were photographed with a Nikon Ci-L light microscope using a digital camera Nikon Ds-Ri2.

### 2.9. Morphometric Evaluation

Two trained investigators (DP and AM) blindly performed all quantitative evaluations. The mean glomerular area (TGA), expressed in square micrometers (μm^2^), was calculated from ten HE-stained sections of each group, measuring twenty glomeruli of the cortical region with the Image J software (National Institute of Health, Bethesda, MD, USA) [50].

Tubular damage was assessed according to a previously described method [18,51]. Briefly, twenty micrographs from PAS-stained sections of each group were studied and evaluated according to the following score: 0 = undamaged; 0.5 = reduction of the brush border with or without interstitial edema; 1 = lower tubular epithelial cells with or without interstitial edema; 2 = incomplete presence of the tubular epithelium with or without interstitial edema; 3 = tubular necrosis with interstitial edema.

A morphometric study to quantitatively assess IL-1β and Nrf2 expression was also performed with ImageJ software. The RGB color images were converted in 32-bit grayscale images, using the function Image > type > 32-bit. A unit area (UA) of 200 × 200 μm, including only tubules, was selected and the grayscale values of twenty UAs of each group were calculated in optical units (OU) from 0 = black to 255 = white. With this method, a higher expression of IL-1β and Nrf2 corresponded to darker images and was reported as lower values in the 0–255 grayscale; a lower expression of IL-1β and Nrf2 was indicated by lighter images, corresponding to higher values on the same scale.

### 2.10. Statistical Analysis

Values are expressed as mean ± standard error (SE). The statistical significance of differences between groups was established using the Student’s *t*-test. The statistical evaluation of differences among groups was performed with ANOVA comparison test. The statistical analysis of histological scores was performed using Mann–Whitney U test with Bonferroni correction. A *p*-value of ≤ 0.05 was considered statistically significant.

## 3. Results

### 3.1. Effects of Nutraceuticals on Urea Nitrogen and Creatinine Levels

Levels of urea nitrogen and creatinine are often employed as biomarkers for the evaluation of kidney function. In our study, no significant differences in urea nitrogen and creatinine levels were observed in the serum of all control groups; therefore, only one value is indicated for controls (Table 2). Urea nitrogen and creatinine levels were significantly increased in CdCl_2_-challenged mice, compared to control groups (*p* < 0.05). In CdCl_2_-challenged animals co-treated with all tested nutraceuticals, urea nitrogen and creatinine levels were lower than CdCl_2_ + vehicle group (*p* < 0.05), being similar to control mice in those treated with BJe at the dose of 40 mg/kg and with both associations (CdCl_2_ + Cur 50 mg/kg + Re 20 mg/kg + BJe 20 mg/kg and CdCl_2_ + Cur 100 mg/kg + Re 20 mg/kg + BJe 40 mg/kg).

### 3.2. Effects of Nutraceuticals on GSH and GPx Levels

It is known that in biological system, Cd induces oxidative stress by intracellular GSH depletion or by inhibiting antioxidant enzymes, such as GPx. The results of our study, suggested that as for urea nitrogen and creatinine content, no significant differences in GSH and GPx levels were present between all control groups; therefore, a single value is indicated for controls (Table 3). A significant decrease in GSH and GPx levels was observed in CdCl_2_-challenged mice (*p* < 0.05). In animals treated with Cur, Re and BJe and challenged with CdCl_2_, GSH and GPx levels were higher compared to controls mice, being almost superimposable to control mice in those treated with BJe (40 mg/kg) and with both associations (CdCl_2_ + Cur 50 mg/kg + Re 20 mg/kg + BJe 20 mg/kg and CdCl_2_ + Cur 100 mg/kg + Re 20 mg/kg + BJe 40 mg/kg) (Table 3).

### 3.3. Effects of Nutraceuticals on Apoptotis-Related Genes

**Figure 2:**
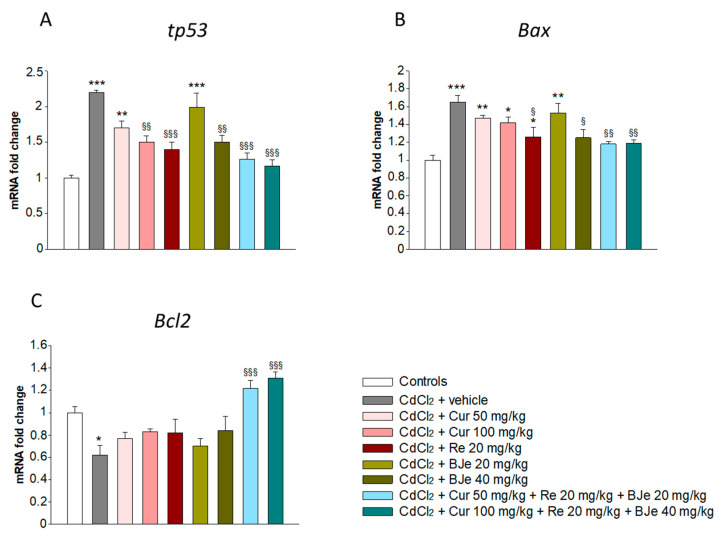
Real-time PCR analysis for *tp53* (**A**), *Bax* (**B**) and *Bcl2* (**C**). * *p* < 0.05, ** *p* < 0.01 and *** *p* < 0.001 vs. control mice; ^§^ *p* < 0.05, ^§§^ *p* < 0.01 and ^§§§^ *p*< 0.001 vs. CdCl_2_-treated mice.

It is well-recognized that both oxidative and inflammatory pathways started by Cd may induce apoptosis, which plays a key role in Cd-caused nephrotoxicity. Therefore, in our study we evaluated the involvement of apoptosis-related genes in kidney of mice exposed to CdCl_2_ with or without nutraceuticals. No significant difference was observed in mRNA levels of *tp53*, *Bax*, and *Bcl2* among the control groups, therefore only one value is indicated for controls. Important changes in *tp53*, *Bax* and *Bcl2* genes were observed in the kidneys of CdCl_2_-treated mice compared to control groups. Moreover, the upregulation of *tp53* and *Bax* found in CdCl_2_-challenged mice were hampered by Cur, Re, and BJe, as well as by their associations (Figure 2). In addition, the downregulation of *Bcl2* observed in CdCl_2_-subjected animals were significantly counteracted by Cur, Re, and BJe, along with their associations (Figure 2).

### 3.4. Effects of Nutraceuticals on Nos2 and Il1b Gene Expression

**Figure 3:**
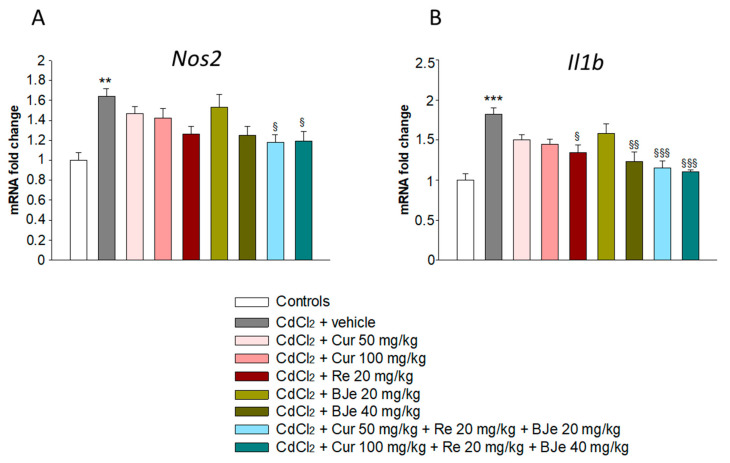
Real-time PCR analysis of *Nos2* (**A**) and *Il1b* (**B**). ** *p* < 0.01 and *** *p* < 0.001 vs. control mice; ^§^ *p* < 0.05, ^§§^ *p* < 0.01 and ^§§§^ *p* < 0.001 vs. CdCl_2_-treated mice.

The high production of iNOS exerts nephrotoxic injury, which in turn can be responsible for the initiation and progression of kidney tubulo-interstitial illness. Therefore, we evaluated the genes level of *Nos2*, along with *Il1b*, a key marker of inflammation, that represent a pathogenic event associated with Cd exposure. As shown in Figure 3, a significant upregulation of *Nos2* and *Il1b* were observed in CdCl_2_-challenged mice when compared to control animals. Notably, a reduction of their mRNA levels was found in the kidneys of all groups treated with Cur, Re and BJe compared to those from Cd-challenged mice. This reduction was significant in the kidney of mice treated with Re 20 mg/kg and BJe 40 mg/kg, reaching the maximum extent when animals were treated with both Cur 50 mg/kg + Re 20 mg/kg + BJe 20 mg/kg and Cur 100 mg/kg + Re 20 mg/kg + BJe 40 mg/kg (Figure 3).

### 3.5. Effects of Nutraceuticals on Nrf2, Nqo1 and Hmox1 Gene Expression

**Figure 4:**
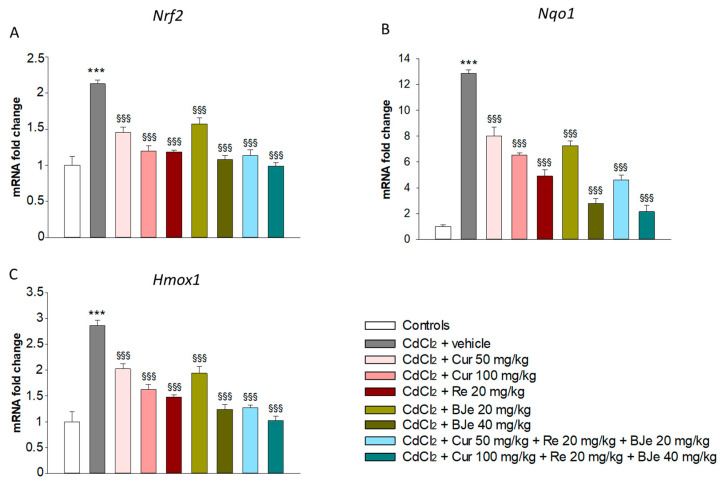
Real-time PCR analysis of *Nrf2* (**A**), *Nqo1* (**B**) and *Hmox1* (**C**). *** *p* < 0.001 vs. control mice; ^§§§^ *p* < 0.001 vs. CdCl_2_-treated mice.

Nrf2 is a crucial transcription factor that induces the expression of cellular defense enzymes to counteract oxidative stress, such as *Nqo1* and *Hmox1*. Data of Real-time PCR analyses showed a significant up-regulation of *Nrf2*, *Nqo1* and *Hmox1* gene expression in CdCl_2_-challenged mice when compared to control animals (Figure 4). In particular, the exposure to CdCl_2,_ enhanced the mRNA levels of *Nrf2*, *Nqo1* and *Hmox1* genes in the kidney mice up to 2.1-, 12.8- and 2.8-fold, respectively (*p* < 0.001; Figure 3). Contrariwise, a significant decrease of *Nrf2*, *Nqo1* and *Hmox1* mRNA levels was found in the kidneys of all experimental groups of animals treated with all doses of Cur, Re and BJe compared to those from Cd-challenged mice. This fall was particularly evident in the kidneys of mice treated with the highest dose of BJe 40 mg/kg (*p* < 0.001) and both Cur 50 mg/kg + Re 20 mg/kg + BJe 20 mg/kg (*p* < 0.001) and Cur 100 mg/kg + Re 20 mg/kg + BJe 40 mg/kg associations (*p* < 0.001) (Figure 4).

### 3.6. Histological and Morphometric Evaluation

**Figure 5:**
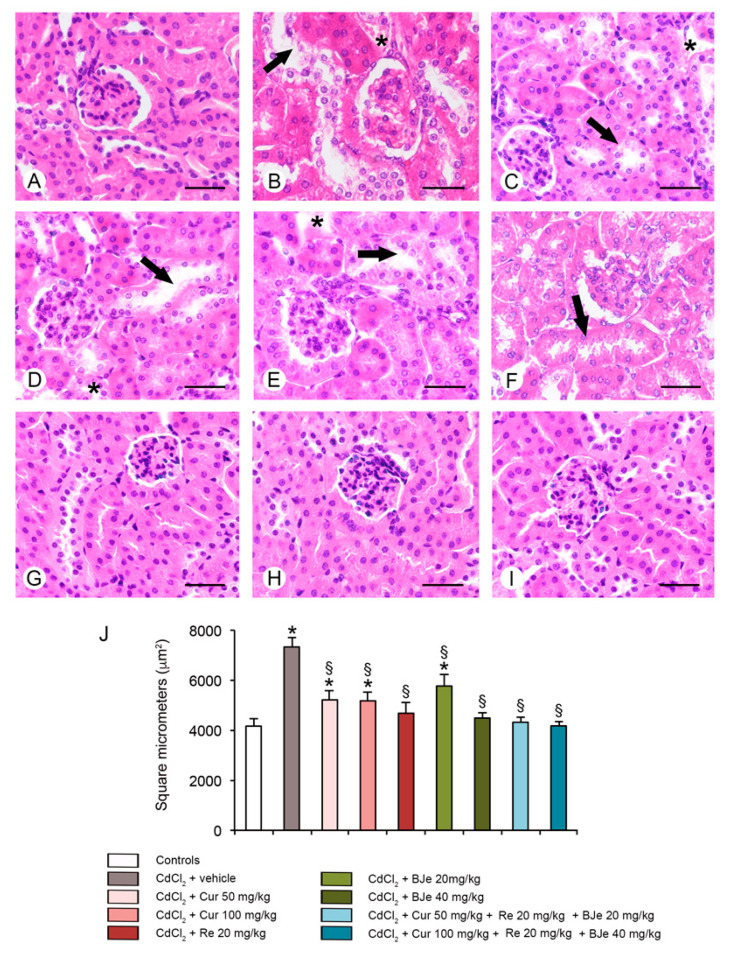
Histological organization of the kidneys examined with hematoxylin-eosin stain. (**A**) Control mice. The normal structure of both tubules and glomeruli is evident. (**B**) Mice challenged with CdCl_2_. An irregular organization of glomeruli, evident changes of the tubular epithelium (arrow) and interstitial edema (asterisk) are present. (**C**,**D**) Mice challenged with CdCl_2_ and treated with Cur at 50 or 100 mg/kg. Tubules show epithelial cells with cytoplasmic changes (arrow) and interstitial edema (asterisk). (**E**) Kidney of CdCl_2_ plus Re treated mice. Some tubules are lined by epithelial cells with altered morphology (arrow). Extra-tubular edema is reduced (asterisk). (**F**) Kidney of CdCl_2_ plus BJe at 20 mg/kg treated mice. The number of damaged tubules (arrow) is increased if compared to Re-treated mice. (**G**–**I**) kidneys from mice treated with CdCl_2_ plus BJe alone at 40 mg/kg and with both the associations Cur 50 mg/kg + Re 20 mg/kg + BJe 20 mg/kg and Cur 100 mg/kg + Re 20 mg/kg + BJe 40 mg/kg. In all groups, the tubules have a normal structure and no edema is present in the interstitial compartment (**J**) quantitative evaluation of the mean glomerular area in the different groups of mice. * *p* < 0.05 vs. control; ^§^ *p* < 0.05 vs. CdCl_2_-treated mice. Scale bar: 50 µm.

**Figure 6:**
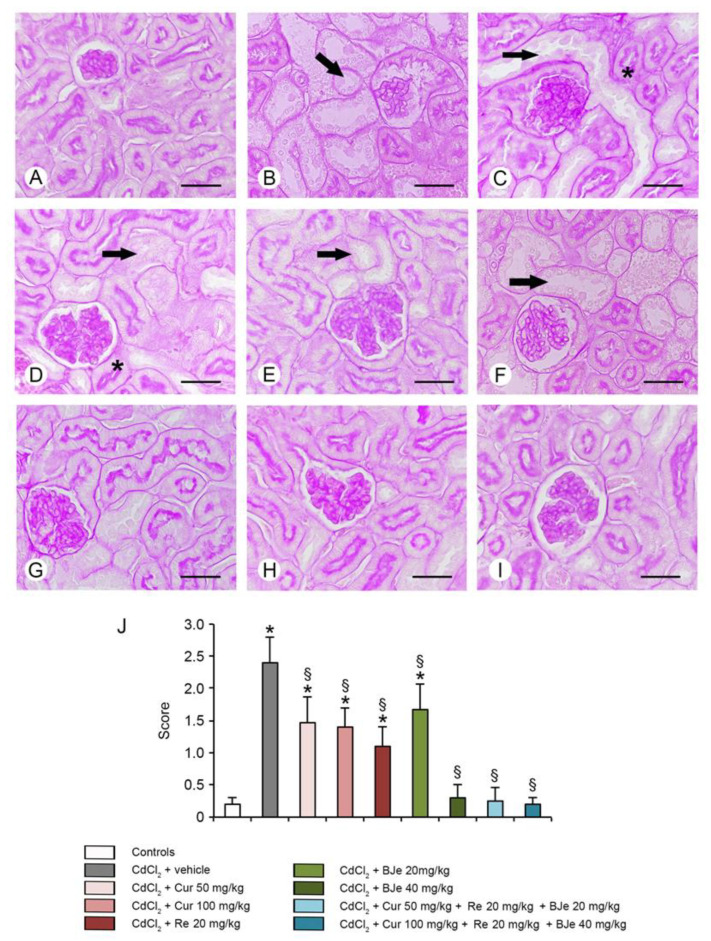
Tubular brush border of the kidneys treated with PAS stain. (**A**) In control mice the proximal tubules show a regular and evident brush border. (**B**) In CdCl_2_-challenged mice, the brush border is particularly thin or absent (arrow) and structural changes are present in the tubular epithelium. (**C**,**D**) Mice challenged with CdCl_2_ and treated with Cur at 50 or 100 mg/kg. Some tubules show lack of staining with PAS (arrow) and damaged epithelial cells, other show a normal structure (asterisk). (**E**) Kidney of CdCl_2_ plus Re treated mice. The number of tubules lined by epithelial cells with altered morphology is reduced (arrow). (**F**) Kidney of CdCl_2_ plus BJe at 20 mg/kg treated mice. Tubules negative to PAS stain and with damaged cells (arrow) is increased if compared to Re treated mice. (**G**–**I**) Kidneys from mice treated with CdCl_2_ plus BJe alone at 40 mg/kg and with both the extract associations Cur 50 mg/kg + Re 20 mg/kg + BJe 20 mg/kg and Cur 100 mg/kg + Re 20 mg/kg + BJe 40 mg/kg. In all groups, the brush border and the tubules have normal organization. (**J**) Tubular damage score based on the brush border behavior. * *p* < 0.05 vs. control; ^§^ *p* < 0.05 vs. CdCl_2_ plus vehicle. Scale bar: 50 µm.

For histological evaluation, kidney sections stained with HE and PAS were examined (Figure 5 and Figure 6). In kidney sections of all control groups stained with HE, glomeruli and tubules showed normal histological organization (a single micrograph is provided for all controls; Figure 4A). In CdCl_2_-challenged mice, glomeruli with enlarged Bowman’s spaces, tubules with epithelial damages and interstitial edema were observed (Figure 5B). In mice challenged with CdCl_2_ with Cur at both doses, cellular lesions and interstitial edema were reduced (Figure 5C,D). In re-treated CdCl_2_-challenged mice, a good preservation of glomeruli was observed, even if some damaged tubules were present (Figure 5E). In CdCl_2_-treated mice, BJe alone at both doses of 20 mg/kg or 40 mg/kg, showed protection of both glomeruli and tubules, even if to a different extent (Figure 5F,G). Similarly, both associations (Cur 50 mg/kg + Re 20 mg/kg + BJe 20 mg/kg and Cur 100 mg/kg + Re 20 mg/kg + BJe 40 mg/kg), demonstrated a well-evident protective action against CdCl_2_, being glomerular and tubular morphology close to normal (Figure 5H,I). The morphometric evaluation of the glomerular area demonstrated a significant higher surface in CdCl_2_-challenged mice, when compared to control groups and a progressive reduction in all the examined groups, with the exception of BJe at lower dose (Figure 5J). When kidney sections were stained with PAS, the proximal tubules of all control groups showed a regular and well stained brush border (a single micrograph is provided for controls; Figure 6A). On the contrary, in CdCl_2_-challenged mice, the brush border was thin or absent (Figure 6B). In CdCl_2_-challenged mice administered with Cur at both doses, tubules showed a more PAS-positive brush border if compared to CdCl_2_ alone (Figure 6C,D). A similar pattern was observed in Re-treated animals (Figure 6E). In mice treated with BJe, the lower dose (20 mg/kg) showed a reduced PAS-positivity, while the brush border was better preserved with the higher dose (40 mg/kg) (Figure 6F,G). The morphological pattern in mice treated with both the associations (Cur 50 mg/kg + Re 20 mg/kg + BJe 20 mg/kg and Cur 100 mg/kg + Re 20 mg/kg + BJe 40 mg/kg) was close to normal (Figure 6H,I). The morphometric evaluation of the tubular damage demonstrated significantly higher scores in CdCl_2_-challenged mice, when compared to control groups, and a progressive reduction of the scores in all the examined groups, with the exception of BJe at lower dose (Figure 6J).

### 3.7. Immunohistochemistry for IL-1β and Nrf2

**Figure 7:**
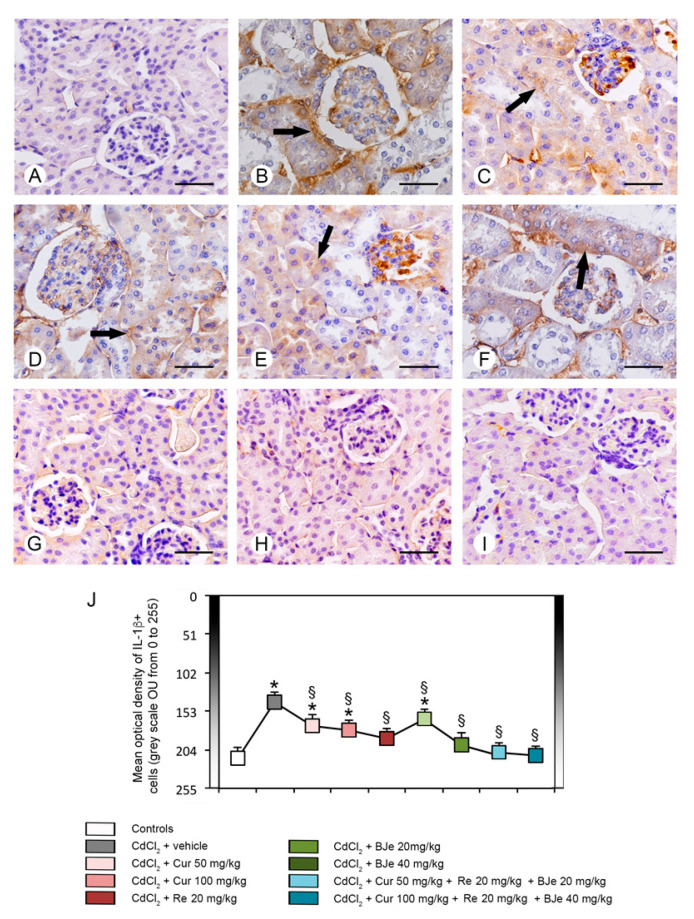
Immunohistochemical localization of IL-1β in the kidneys. (**A**) In all control groups, no IL-1β immunoreactivity can be demonstrated. (**B**) In CdCl_2_ plus vehicle-treated mice, nearly all tubules showed a strong IL-1β immunoreactivity (arrow). (**C**–**E**) In mice treated with CdCl_2_ plus both doses of Cur and with CdCl_2_ plus Re, a moderate IL-1β immunoreactivity was present in some tubules (arrow). (**F**) In CdCl_2_ plus BJe at the lower dose challenged mice, IL-1β immunoreactivity (arrow) was milder if compared to CdCl_2_ alone treated mice, but higher respect to Cur and Re. (**G**–**I**) In mice treated with CdCl_2_ plus BJe at 40 mg/kg and with CdCl_2_ plus both associations, IL-1β immunoreactivity was absent, similar to controls. (**J**) Morphometric results for IL-1β expression. Data are expressed in Optical Units/Unit Area (OU/UA) (from 0 = black to 255 = white). * *p* < 0.05 vs. control; ^§^ *p* < 0.05 vs. CdCl_2_ plus vehicle. Scale bar: 50 µm.

IL-1β immunoreactivity was undetectable in all control groups; therefore, a single micrograph is provided as representative of all controls (Figure 7A). In CdCl_2_ plus vehicle treated mice, almost all tubules displayed a strong IL-1β immunoreactivity (Figure 7B). In mice treated with CdCl_2_ plus both doses of Cur or CdCl_2_ plus Re, a moderate IL-1β immunoreactivity was present in some tubules (Figure 7C–E). In CdCl_2_ plus BJe at the lower dose challenged mice, IL-1β immunoreactivity was milder if compared to CdCl_2_ alone treated mice, but higher in respect to Cur and Re (Figure 7F). In mice treated with CdCl_2_ plus BJe at 40 mg/kg and with CdCl_2_ plus both associations, IL-1β immunoreactivity was similar to controls (Figure 7G–I). The quantitative assessment of IL-1β expression revealed significant lower values (high immunoreactivity) in the 0–255 grayscale in CdCl_2_ treated mice vs. controls and a progressive reduction of the optical density (low immunoreactivity), milder only for BJe at the lower dose (Figure 7J).

**Figure 8:**
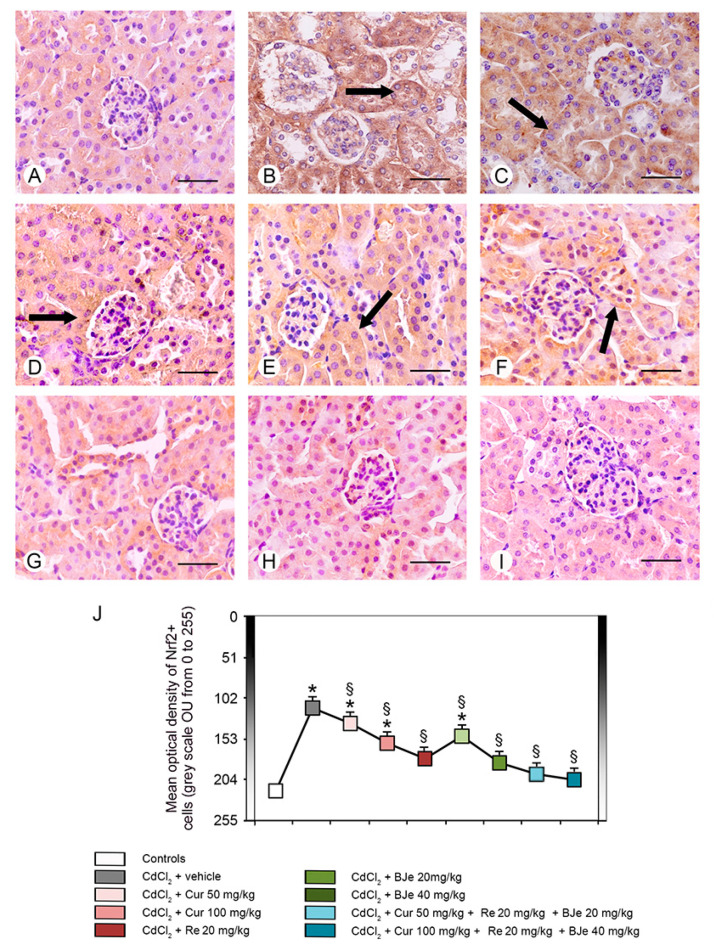
Immunohistochemical localization of Nrf2 in the kidneys. (**A**) In all control groups, Nrf2 immunoreactivity is particularly strong in the tubular wall (arrow). (**B**) In CdCl_2_ plus vehicle-treated mice, no Nrf2 immunoreactivity is present. (**C**–**E**) In mice treated with CdCl_2_ plus both doses of Cur and with CdCl_2_ plus Re, Nrf2 shows a moderate positivity in some tubules (arrow). (**F**) In mice challenged with CdCl_2_ plus BJe at the lower dose, Nrf2 immunoreactivity was lower (arrow) when compared to Cur and Re. (**G**–**I**) Mice treated with CdCl_2_ plus BJe at 40 mg/kg and with CdCl_2_ plus both associations: Nrf2 immunoreactivity is high (arrow), similar to controls. (**J**) Morphometric results for Nrf2 expression. Data are expressed in optical units/unit area (OU/UA) (from 0 = black to 255 = white). * *p* < 0.05 vs. control; ^§^ *p* < 0.05 vs. CdCl_2_ plus vehicle. Scale bar: 50 µm.

Nrf2 immunoreactivity was high in all control groups; therefore, a single micrograph is provided to show the morphological pattern of all controls (Figure 8A). In CdCl_2_ plus vehicle treated mice, Nrf2 immunoreactivity was absent nearly in all the tubules (Figure 8B). When mice were treated with CdCl_2_ plus both doses of Cur or CdCl_2_ plus Re, Nrf2 immunoreactivity showed a moderate pattern in some tubules (Figure 8C–E). In mice challenged with CdCl_2_ plus BJe at the lower dose, Nrf2 immunoreactivity was lower if compared to Cur and Re (Figure 8F). Instead, mice treated with CdCl_2_ plus BJe at 40 mg/kg and plus both associations demonstrated Nrf2 immunoreactivity similar to controls (Figure 8G–I). The quantitative assessment of Nrf2 tubular expression demonstrated significant higher values (low immunoreactivity) in the 0–255 grayscale in CdCl_2_ challenged mice versus controls and lower values (high immunoreactivity) in all groups, with the exception of CdCl_2_ plus BJe at the lower dose of 20 mg/kg (Figure 8J).

## 4. Discussion

Cadmium pollution is rising worldwide because of intensified industrial activities, that have increased its availability, and its significant environmental persistency. The role of cadmium in physiological processes is not yet fully understood; however, it competes with other essential metal ions, thereby disrupting cell functions. It causes damage to various organs in mammals by causing teratogenicity, genotoxicity, osteoporosis, neurotoxicity and nephrotoxicity [52].

In this study, we observed high values of serum creatinine and blood urea nitrogen of mice exposed to Cd, demonstrating the kidney injury induced by this metal. Levels of these parameters, as well as urine analysis, are often employed as biomarkers for the evaluation of kidney function, despite their limitations for the detection of early stages of kidney diseases [53]. Urea is the major nitrogenous end product of protein and amino acid catabolism. Increased blood urea nitrogen is acknowledged to be associated with kidney disease or failure, blockage of the urinary tract by kidney stones, congestive heart failure, dehydration, fever, shock and bleeding in the digestive tract [54]. Creatinine is a nitrogenous compound formed by creatine and phosphocreatine during muscular metabolism and primarily eliminated through glomerular filtration. It is commonly used as measure of kidney function. We demonstrated that Cur, Re and BJe administration, alone or in combination, reversed Cd-induced nephrotoxicity by reducing the elevated levels of creatinine and blood urea nitrogen in the serum of Cd-treated mice reaching the maxim effect with BJe 40 mg/kg and with both association Cur 50 mg/kg + Re 20 mg/kg + BJe 20 mg/kg and Cur 100 mg/kg + Re 20 mg/kg + BJe 40 mg/kg. This finding was in accordance with previous research showing the improvement of nephrotoxicity by Cur and Re in other experimental models [55,56].

As known, cadmium induces oxidative stress by altering the pro-oxidant/antioxidant balance in animal tissues. In biological systems, Cd does not undergo redox reactions, but it can induce oxidative stress by intracellular GSH depletion [57] or by inhibiting antioxidant enzymes, such as GPx, interacting with their thiol groups [58]. Previous studies demonstrated that GSH depletion enhances Cd-induced hepatotoxicity and that GSH precursor N-acetylcysteine prevents Cd-induced oxidative stress and toxicity in the liver and brain of Cd-exposed rats [59]. These data have been recently confirmed by Zhang and co-workers that demonstrated the protective effects of Re in Cd-induced nephrotoxicity, mitigating GSH depletion and restoring the activity of antioxidant enzymes [60].

In this study, administration of Cd led to oxidative stress which is evidenced by reduced levels of the antioxidant enzymes GPx and GSH that was significantly restored by Cur, Re and BJe, alone or in association.

Furthermore, Cd induces the inducible form of nitric oxide synthase (iNOS), responsible for nitrosative stress. The level of iNOS is very low in healthy kidney [61] and, when produced in large amount, it exerts nephrotoxic injury, with proximal tubules and glomeruli dysfunction in different experimental models, such as renal ischemia/reperfusion [62]. CdCl_2_-treated mice showed an enhanced expression of iNOS in renal tissue compared to controls, which could be related to the generation of ROS, secondary to the structural lesions of tubular epithelial cells. In our study, we observed that both the combination of Cur 50 mg/kg + Re 20 mg/kg + BJe 20 mg/kg and Cur 100 mg/kg + Re 20 mg/kg + BJe 40 mg/kg showed a significant positive action against iNOS expression.

Inflammation is a key pathogenic event associated with Cd exposure [63,64], which is responsible of the initiation and progression of kidney tubulo-interstitial illness [18]. It amplifies the expression of pro-inflammatory and transcriptional factors including tumor necrosis factor alfa (TNF-α), IL-1β and iNOS. In our study, both the expression and the immunohistochemical analysis of IL-1β after Cd challenge were increased, thus explaining its detrimental role in the proximal tubules. Recently, we and other demonstrated the efficacy of several natural extracts and compounds in modulating inflammatory process induced by Cd challenge through the reduction of inflammatory markers expression in the kidney [17,18,61,65,66]. In this study, all the examined nutraceuticals and both the associations reduced the expression and the immunohistochemical analysis of IL-1β decreasing the inflammatory damages induced by Cd. These biochemical and molecular data were confirmed by the histopathological examinations, which demonstrated an increased glomerular area, a reduced PAS stain of the proximal tubules brush border, the presence of tubular cells lesions and of interstitial edema, as already demonstrated in previous observations [17,18]. We demonstrated that Cur and Re reduced cellular lesions and interstitial edema, as well as we observed a more PAS-positive brush border of tubules if compared to CdCl_2_. Moreover, BJe alone at both doses, showed protection of both glomeruli and tubules, despite to a different extent. However, BJe at lower dose did not reduce the surface of glomerular area. In addition, BJe at the lower dose showed a reduced PAS-positivity, while the brush border was better preserved with the higher dose. Interestingly, both the associations (Cur 50 mg/kg + Re 20 mg/kg + BJe 20 mg/kg and Cur 100 mg/kg + Re 20 mg/kg + BJe 40 mg/kg), demonstrated a well evident protective action against CdCl_2_, being glomerular and tubular morphology close to normal. Moreover, the morphological pattern in mice treated with both the associations was similar to that of the controls. The morphometric evaluation of the tubular damage demonstrated significantly higher scores in CdCl_2_-challenged mice, when compared to control groups, and a progressive reduction of the scores in all the examined groups, with the exception of BJe at lower dose.

It is well-acknowledged that both oxidative and inflammatory pathways triggered by Cd may activate apoptosis which plays a pivotal role in Cd-caused nephrotoxicity [67]. Previous studies reported that the occurrence of apoptosis involves p53 and its downstream targets, and that the anti- and pro-apoptotic members of the Bcl-2 family are crucial effectors for p53-regualted apoptosis [68]. In this study, we found that Cd exposure augmented apoptosis in the kidneys. In mice, it was demonstrated that Bcl-2 expression is down-regulated and Bax up-regulated after exposure to Cd [69]. Similar results, together with the upregulation of p53, were observed in our research, thus confirming an important role of Cd in triggering apoptosis. In this context, many natural substances have shown a promising role in positively modulating the apoptotic pathways after Cd treatment, such as selenium [70], *Potentilla anserina* polysaccharide [71], betulinic acid [72], myo-inositol [18], vitamin E [73], quercetin [66]. In our study, we observed that Cur, Re and BJe protected against Cd-induced apoptosis, even if both the association Cur 50 mg/kg + Re 20 mg/kg + BJe 20 mg/kg and Cur 100 mg/kg + Re 20 mg/kg + BJe 40 mg/kg) provided better defense against apoptotic process.

In the last decade, several studies suggested that disruption of the Nrf2 signaling pathway was involved in several kidney diseases [74,75,76]. Nrf2 is a crucial transcription factor that plays a pivotal role in inducing the expression of cellular defense enzymes to counteract oxidative stress. Physiologically, Nrf2 assembles cytosolic Kelch-like ECH associated protein-1 (Keap1). In response to the oxidative stress injury, Nrf2 translocates into the nucleus, binding to a highly conserved enhancer antioxidant responsive element (ARE) and regulating transcription of a different of phase II metabolism and detoxification genes, such as heme oxygenase 1 (HO-1) and NQO1. Briefly, the Nrf2-regulated antioxidant response serves to contrast oxidative injury and to preserve intracellular redox homeostasis. In this study, we observed a significant up-regulation of Nrf2 gene in kidney of Cd-treated mice. As consequence, the expression levels of downstream Nrf2 signaling genes *Hmox1* and *Nqo1* were up-regulated. Our results are clearly consistent with previous research, wherein the administration of Cd significantly increased the expression of Nrf2 and further upregulated the expression of downstream phase II detoxification enzymes [77,78]. This result further confirms that Nrf2-ARE signaling is a crucial regulator for cells to maintain the oxidant/antioxidant balance. Finally, as expected, the results showed that pre-treatment with BJe, Cur or Re alone, or in combination (Cur 50 mg/kg + Re 20 mg/kg + BJe 20 mg/kg and Cur 100 mg/kg + Re 20 mg/kg + BJe 40 mg/kg) inhibited the Cd-activated Nrf2 signaling pathway.

## 5. Conclusions

In conclusion, for the first time, the results of our study suggest that BJe reduces CdCl_2_-induced oxidative damage in the kidneys of challenged mice. It significantly improved the impaired renal functionality, along with reducing morphological changes of glomeruli and proximal tubules, which are known as key targets for Cd nephrotoxicity. Moreover, BJe restored GSH content and GPx activity, counteracted *Nos2* and *Il1b* over-expression and hampered kidney damage through a mechanism involving its anti-apoptotic activity. Finally, BJe was able to modulate the Nrf2 pathway and its downstream signaling genes *Hmox1* and *Nqo1*, increased by CdCl_2_.

Generally, each nutraceutical is employed alone to achieve the desired outcome; however, in the recent past, we as others [79,80,81] have highlighted the relevance of a multitarget pharmacological strategy to deal with a disease. Indeed, pathologies are multifactorial events which hence require the necessity to aim at different targets simultaneously.

On this line, we indicated the effectiveness of BJe, likely due to being a phytocom-plex, and that also the association with Cur and Re, well-known bioactive principles, amplify the protective effect of BJe, thus being the first to focus on the combination of nutraceuticals to test their effects to protect renal functions after exposition to environmental toxicants. Our results need to be also proven in clinical studies to definitively assure our statements, although they offer a solid foundation for a new strategy to fight heavy metal toxicity.

## Figures and Tables

**Table 1:** Oligonucleotide primers used for the quantitative Real-time PCR analyses.

Gene	Protein	NCBI Reference Sequence	Primer Sequence
***Actb***	Beta-actin	NM_007393.5	F: GCTGTGCTATGTTGCTCTA R: TCGTTGCCAATAGTGATGA
***Bax***	Apoptosis regulator BAX	NM_007527.3	F: GCGAATTGGAGATGAACT R: CAGTTGAAGTTGCCATCA
***Bcl2***	Apoptosis regulator Bcl-2	NM_009741.5	F: GTGGAGGAACTCTTCAGG R: TGACATCTCCCTGTTGAC
***Hmox1***	Heme oxygenase 1	NM_002133.3	F: CGCCTTCCTGCTCAACAT R: ACGAAGTGACGCCATCTG
***Il1b***	Interleukin-1 beta	NM_008361.4	F: ATCTATACCTGTCCTGTGTAATGA R: GCTTGTGCTCTGCTTGTG
***Nos2***	Nitric oxide synthase, inducible	NM_010927.4	F: GAGCGAGTTGTGGATTGT R: GCAGCCTCTTGTCTTTGA
***Nqo1***	NAD(P)H dehydrogenase [quinone] 1	NM_009706.5	F: TCAGTATCCTTCCGAGTCATC R: TCAAACCAGCCTTTCAGAAT
***Nrf2***	Nuclear factor erythroid 2-related factor 2	NM_010902.4	F: CAGCACCTTGTATCTTGAAGT R: GCAACACATTGCCATCTCT
***Tp53***	Tumor suppressor p53	NM_001127233.1	F: TGGAAGACAGGCAGACTT R: ACTTGTAGTGGATGGTGGTA

**Table 2:** Effects on urea nitrogen and creatinine levels in control mice, in cadmium chloride (CdCl_2_; 2 mg/kg i.p.) plus vehicle exposed ones and in those exposed to CdCl_2_ (2 mg/kg i.p.) co-administered with curcumin (Cur), resveratrol (Re), bergamot juice extract (BJe), or their combinations. All values are expressed as mean ± SE; *n* = 7 animals for each group.

	Urea Nitrogen (mg/dL)	Creatinine (mg/dL)
Controls	14.5 ± 1.7	0.68 ± 0.1
CdCl_2_ + vehicle	41.2 ± 3.6 ^a^	1.51 ± 0.33 ^a^
CdCl_2_ + Cur 50 mg/kg	32.2 ± 2.9 ^a,b^	1.24 ± 0.4 ^a,b^
CdCl_2_ + Cur 100 mg/kg	30.3 ± 2.5 ^a,b^	1.21 ± 0.33 ^a,b^
CdCl_2_ + Re 20 mg/kg	26.6 ± 3.1 ^a,b^	1.02 ± 0.37 ^a,b^
CdCl_2_ + BJe 20 mg/kg	34.3 ± 2.1 ^a,b^	1.29 ± 0.28 ^a,b^
CdCl_2_ + BJe 40 mg/kg	18.3 ± 1.9 ^b^	0.77 ± 0.29 ^b^
CdCl_2_ + Cur 50 mg/kg + Re 20 mg/kg + BJe 20 mg/kg	15.9 ± 1.6 ^b^	0.73 ± 0.18 ^b^
CdCl_2_ + Cur 100 mg/kg + Re 20 mg/kg + BJe 40 mg/kg	14.7 ± 1.9 ^b^	0.71 ± 0.15 ^b^

^a^*p* < 0.05 vs. controls; ^b^ *p* < 0.05 vs. CdCl_2_ + vehicle.

**Table 3:** Effects on glutathione (GSH) content and glutathione peroxidase (GPx) activity in control mice, in cadmium chloride (CdCl_2_; 2 mg/kg i.p.) plus vehicle exposed mice and in mice exposed to CdCl_2_ (2 mg/kg i.p.) co-administered with curcumin (Cur), resveratrol (Re), bergamot juice extract (BJe), or their combinations. All values are expressed as mean ± SE; *n* = 7 animals for each group.

	GSH (μmol/g of Tissue)	GPx (nmol/min per mg of Protein)
Controls	65 ± 4	34.6 ± 1.9
CdCl_2_ + vehicle	47 ± 5 ^a^	16.3 ± 1.6 ^a^
CdCl_2_ + Cur 50 mg/kg	53 ± 3 ^a^	21.4 ± 0.8 ^a^
CdCl_2_ + Cur 100 mg/kg	54 ± 6 ^a,b^	22.7 ± 0.7 ^a,b^
CdCl_2_ + Re 20 mg/kg	57 ± 4 ^a,b^	26.6 ± 1.1 ^a,b^
CdCl_2_ + BJe 20 mg/kg	51 ± 3 ^a,b^	19.5 ± 1.2 ^a,b^
CdCl_2_ + BJe 40 mg/kg	59 ± 5 ^b^	30.3 ± 0.4 ^b^
CdCl_2_ + Cur 50 mg/kg + Re 20 mg/kg + BJe 20 mg/kg	62 ± 6 ^b^	32.2 ± 1.1 ^b^
CdCl_2_ + Cur 100 mg/kg + Re 20 mg/kg + BJe 40 mg/kg	64 ± 5 ^b^	34.1 ± 1.1 ^b^

^a^*p* < 0.05 vs. controls; ^b^ *p* < 0.05 vs. CdCl_2_ + vehicle.

## Data Availability

The datasets generated for this study are available on request to the corresponding author.
